# The impact of the Italian guidelines on antibiotic prescription practices for acute otitis media in a paediatric emergency setting

**DOI:** 10.1186/s13052-015-0144-4

**Published:** 2015-05-07

**Authors:** Silvia Palma, Cristiano Rosafio, Cinzia Del Giovane, Viviana Dora Patianna, Laura Lucaccioni, Elisabetta Genovese, Paolo Bertolani, Lorenzo Iughetti

**Affiliations:** Audiology Unit, Health Local Unit of Modena, Modena, Italy; Paediatric Unit, Post Graduate School of Paediatrics, University of Modena and Reggio Emilia, Modena, Italy; Department of Diagnostic, Clinical and Public Health Medicine, University of Modena and Reggio Emilia, Modena, Italy; Audiology, Department of Diagnostic, Clinical and Public Health, University of Modena and Reggio Emilia, Modena, Italy; Paediatric Unit, Department of Medical and Surgical Sciences for Mothers, Children and Adults, University of Modena and Reggio Emilia, Modena, Italy

## Abstract

**Background:**

Acute otitis media (AOM) is one of the most common childhood infectious diseases. The recent Italian Pediatric Guidelines for the treatment of AOM constitutes a step forward in the management of children with uncomplicated AOM. The aim of this study was to evaluate antibiotic prescription patterns for AOM in a Pediatric Emergency Department (PED) after those guidelines were introduced and to assess the relationship between implementation of the “watchful waiting” strategy and the incidence of acute mastoiditis in the PED.

**Methods:**

This retrospective study was conducted between 1st January 2007 to 31st December 2013 at the PED of the University of Modena and Reggio Emilia in Modena (Italy). All children between 0 and 14 years who were examined because of symptoms and/or signs of AOM and acute mastoiditis were enrolled. Pearson’s chi-squared test was used to evaluate if introduction of the Italian Paediatric Guidelines was associated with a reduction in the antibiotic prescription pattern in children with AOM and/or with an increase in mastoiditis frequency.

**Results:**

4,573 (89.4%) patients were included in our analysis, antibiotics were prescribed to 81% cases of the children diagnosed with AOM. The frequency of antibiotic prescribing continued to be stable after the Italian guidelines were introduced (82% versus 81%).

Forty children were admitted to hospital with a diagnosis of acute mastoiditis. Our study did not find any association between the number of cases of acute mastoiditis and the percentage of patients treated with antibiotics; the annual incidence of mastoiditis before and after the new guidelines were published was, in fact, stable.

**Conclusions:**

Despite the diffusion of clinical guidelines recommending a “watchful waiting” approach for children with AOM, the antibiotic prescription rate continues to be high. It appears to be more difficult to impact the percentage of cases for which antibiotics are prescribed than the type of antibiotic that is utilized. In view of these findings, a close follow-up control by the primary care paediatrician or a scheduled follow-up appointment at the PED and incisive campaigns to promote parents’ awareness of proper antibiotic use appear to be warranted.

## Background

Acute otitis media (AOM) is the most common disease for which children receive antibiotics [1]. It has been calculated that about 90% of children between the age of 3 months and 2 years experience an episode of AOM; the peak incidence is between 6 and 12 months of age [1-6]. There has been much debate on the optimal treatment for AOM and the prevention of complications. Inappropriate antibiotic treatment continues, moreover, to pose a significant challenge in primary care [2,7]. While antibiotic resistance has become one of the world’s most pressing public health problems, antibiotic prescription rates for AOM are still high in most countries [8,9]. Most parents, nevertheless, perceive AOM to be a burden for their child and families, particularly in view of the pain and disturbed sleep associated to it [10].

“The observation option” treatment strategy that was endorsed by the American Academy of Pediatrics and which recommends antibiotic treatment for children who remain symptomatic for 72 hours is based on studies demonstrating that AOM heals spontaneously in 80% of cases [11]. The Italian Pediatric Guidelines for AOM diagnosis and prevention that were published in 2010 considered the 2004 Clinical Practice Guideline of the American Academy of Pediatrics (AAP) as a model and a starting point [12]. The Italian guidelines recommend that “antibiotic treatment should be prescribed immediately for severe cases of AOM, for children younger than two years with bilateral AOM, and cases of spontaneous perforation. In all other cases, and in agreement with the parents, it is possible to wait watchfully and to prescribe antibiotic treatment only if the episode worsens or does not improve within 48–72 hours. The main treatment of earache is the systemic administration of appropriate doses of analgesics (paracetamol or ibuprofen)” [12]. Amoxicillin was indicated in the guidelines as the first choice drug in the case of AOM in children at low risk of resistant pathogens [12].

Some have expressed concern, nevertheless, that the observation strategy could lead to a higher rate of acute mastoiditis, the most common severe complication associated with AOM [5,6,9].

The principal objective of this study was to assess the impact of the 2010 Italian guidelines on antibiotic prescribing practice for this infection in the Paediatric Emergency Department (PED) of the Paediatric Unit of the University of Modena. The relationship between the implementation of the watchful waiting policy and the incidence of acute mastoiditis in the PED was also evaluated.

## Methods

This retrospective study was conducted between 1st January 2007 to 31st December 2013 in the PED of the Paediatric Unit of the University of Modena, an academic tertiary care pediatric medical center.

As this work represents an appraisal of PED practices before and after Italian guidelines were introduced, an institutional review board approval was not necessary. All of the parents gave consent to collect and analyse data contained in their children’s charts.

The key points contained in the Italian guidelines were presented at educational meetings of the Pediatric Unit involving all staff physicians. The entire document was, moreover, freely accessible on the websites of the Italian Society of Pediatric Otolaryngology (www.siop.it) and of the Italian Society of Pediatrics (www.sip.it).

A trained staff physician screened the charts of the PED for data on the children (aged 0 to 14 years) who were possible candidates for inclusion in the study. “Acute otitis media”, “otitis”, “otorrhea”, “earache”, “acute mastoiditis” and “suspected mastoiditis” as the discharge diagnosis were the search terms that were utilized. Children with immunodeficiency, cranialfacial malformations, a history of mastoiditis or ear surgery (i.e. tympanoplasty or cochlear implant) were excluded from the study as the guidelines are not applicable in these cases; children with external otitis or whose final diagnosis differed from AOM were excluded. All the charts in which the diagnosis was not clear and any ambiguous data were clarified by discussion between the otorhynolaryngologist and the senior paediatrician and those charts were re-evaluated. The variables and the coding management of the search terms were reviewed by other members of the staff.

All the children presenting at the PED were first evaluated by a paediatrician using a static otoscope; in the event of cerumen, otorrhea or in case of suspected\acute mastoiditis, a consultation with an otolaryngologist was requested. Once the diagnosis was made and treatment was decided, the parents were instructed to consult their family paediatrician within 24-48 hours for an appropriate follow-up.

The information collected were: sex, age, discharge diagnosis (AOM or mastoiditis), type of management, and type of antibiotics prescribed (when available). We categorized the children’s ages into three classes: <2 years, between 2 and 6, older than 6. Depending on the type of treatment recommended, the patients were divided into three groups: children receiving no antibiotic prescription, children receiving an antibiotic prescription, and children receiving the recommendation to continue an antibiotic that had already been prescribed. When the recommendation for the case was not registered, those patients were excluded from the analysis (7 cases). The data registered before and after the publication of the Italian guidelines (2007-2010 versus 2011-2013) were classified and analysed. Categorical data were reported as absolute frequencies and percentages. Pearson’s chi-squared test was used to evaluate if publication of the Italian Paediatric Guidelines was associated with a reduction in antibiotic prescriptions in children with AOM and if the watchful waiting approach was associated with an increase in mastoiditis. The analyses were performed using STATA software v13, and a p-value less than 0.05 was considered statistically significant.

## Results

**Figure 1:**
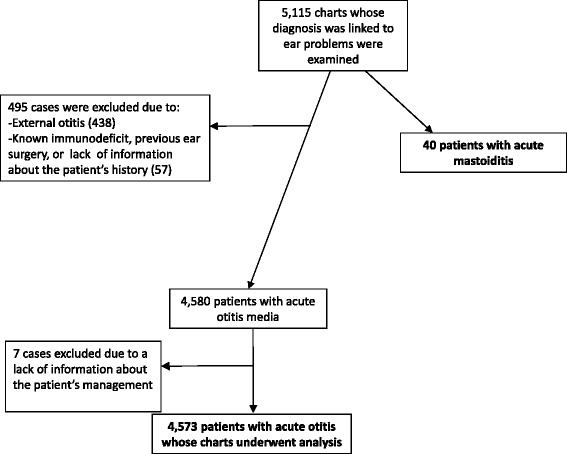
Flowchart of the records of patients examined between 2007 and 2013.

A total of 127,866 children were examined in the PED during the study period; 5,115 of these were originally considered eligible for inclusion (Figure 1). Out of that group*,* 495 were excluded because the final diagnosis was external otitis (438) or because of the presence of predisposing factors (57), and 7 were excluded because of lack of knowledge about the treatment. Of the 4,580 patients who were diagnosed with AOM at discharge, only 4,573 (89.4%) were included in our analysis (Figure 1). Fifty-six percent of the children with AOM included in the analysis were male; 47% <2 years, 38% were between 2 and 6 years, and 15% were older than six.

Excluding 14 children who were already receiving an antibiotic before presenting at the PED, antibiotics were prescribed to 81% cases of the children diagnosed with AOM (Table 1). Analysis of the data revealed that the percentage of antibiotic prescriptions did not vary significantly after the Italian guidelines were introduced (Table 1). The annual antibiotic prescription rate in each age class was likewise analysed and no statistical differences between pre and post rates were found (Table 1).

**Table 1:** **Antibiotics prescribed before and after the guidelines were published**

	**Before the guidelines were introduced (n = 2,692)**	**After the guidelines were introduced (n = 1,867)**	**Total (n = 4,559)**
Period	2007- 2010	2011-2013	
Antibiotics prescribed, n (%)	2,201 (82%)	1,507 (81%)	3,708 (81%)
Amoxicillin, n (%)	716 (33%)	479 (32%)	1,195 (32%)
Amoxicillin–clavulanate, n (%)	1,131 (51%)	755 (50%)	1,886 (51%)
Cefuroxime axetil, n (%)	235 (10%)	159 (11%)	394 (11%)
Macrolides, n (%)	48 (2%)	39 (3%)	87 (2%)
Antibiotics prescribed in the various age classes			
<=2, n (%)	1,135 (86%)	731 (84%)	2,184 (48%)
2-6, n (%)	790 (77%)	540 (78%)	1,715 (38%)
> = 6, n (%)	276 (78%)	236 (77%)	660 (14%)

The most frequently prescribed antibiotic was amoxicillin-clavulanate (51%) and its distribution rate was similar before and after the Italian Guidelines were published (Table 1).

**Figure 2:**
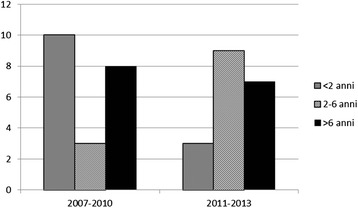
The distribution of mastoiditis across age classes before and after the guidelines were published.

Forty children were admitted to the hospital with a diagnosis of acute mastoiditis over the study period (40/4,580 = 0.9%): 21 (52.5%) between 2007-2010 and 19 (47.5%) between 2011-2013 (Figure 2). Of these, 32.5% were younger than 2 years. In 23 cases (58%) the children had received antibiotic treatment before presenting at the PED. Antibiotics were not prescribed in 43% of the cases of acute mastoiditis before the Italian guidelines were published; they were not prescribed in 42% after they were published; the difference was not statistically significant. The mean number registered per year was 6 cases (min-max = 4-7 in 2009 and 7 in 2013). ***When the number of mastoiditis cases and the annual antibiotic prescription rate was analysed, no relevant difference was found (Table***2***).***

**Table 2:** **Patients presenting at our Pediatric Emergency Service (PED) between 2007 and 2013**

**Year**	**Number of patients presenting at the PED**	**Number (%) of patients with Acute Otitis Media**	**Number (%) of patients with Acute Otitis Media prescribed antibiotics**	**Number (%) of patients with Acute Mastoiditis**
2007	17471	638 (3.7)	513 (80.4)	5 (0.03)
2008	18164	812 (4.5)	656 (80.8)	6 (0.03)
2009	18036	629 (3.5)	535 (85.1)	4 (0.02)
2010	17660	620 (3.5)	497 (80.2)	6 (0.03)
2011	18525	634 (3.4)	495 (78.1)	6 (0.03)
2012	18975	636 (3.4)	522 (82.1)	6 (0.03)
2013	19035	604 (3.2)	490 (81.1)	7 (0.03)

## Discussion

The principal objective of this study was to assess the impact of the Italian Guidelines for the clinical management of AOM in a hospital setting. To our knowledge, this is the first study that has analysed antibiotic prescribing patterns for AOM in patients admitted to an Italian PED. The results demonstrate that a large number of children are still evaluated in a PED for AOM and that the watchful waiting strategy is still far from routine use [13].

According to clinical activity databases, in most countries in which there are national guidelines for the management of AOM, adherence to the guidelines has been poor [9]. Our study found a stable percentage in antibiotic use in the periods before and after the Italian guidelines were introduced in all age classes. We had expected to see a high, stable percentage of antibiotic prescriptions only in very young children as AOM is most frequent in infants between 6 and 13 months of age [14]. These findings could be explained if patients presenting at the PED after the guidelines were introduced had more complex problems with respect to those seen in the past.

It is also possible that parents decide to take their children to the PED in the event that symptoms persist once a watchful waiting strategy has been prescribed by the family paediatrician. While AOM has a low priority at a PED and waiting times can be lengthy, many parents are, nevertheless, very concerned about the infection and the impact it can have on the child’s health. Fear about the adverse consequences of the infection seems to be the main obstacle to implementing the guidelines. Staff paediatricians in the emergency room are probably not convinced that the watchful waiting strategy is an appropriate option for a PED setting and doubts linked to patient follow-up may lead to more cautious therapeutic approaches [15].

As an accurate otological examination is critical for disease recognition, an uncertain diagnosis can also justify the high rate of antibiotic prescription. The initial choice of treatment may also be based on the general impact that the disease produces in each individual patient [16]. Particular attention must, of course, be dedicated to the child’s clinical condition and to such factors as age, signs and symptoms as well as parents compliance: parents who are uninformed about why appropriate use of antibiotics is so important may be resistant to the watch and see guideline [10,17]. One study recently demonstrated that a watchful waiting policy with and without a prescription of antibiotics was tranquilly accepted by both groups of parents of children diagnosed with AOM in an urban PED [18]. This finding confirms that parents can accept this approach if they are provided appropriate explanations.

The objective of containing antibiotic-resistance is one of the aims of the guidelines, an aim that should be shared by physicians and parents alike, and the judicious use of antibiotics remains the key to an optimal management to prevent the complications of acute otitis media.

The risk of increasing multi-drug-resistant bacteria is well known. Our study showed that the majority of children attending our PED, regardless of age, were prescribed broad–spectrum antibiotics such as amoxicillin-clavulanic acid and cefuroxime. But similar studies have demonstrated that the kind of antibiotics prescribed can rapidly change under the pressure of new recommendations; this means that every effort to follow to the guidelines should continue to be made [19-21].

Acute mastoiditis is the most common severe complication associated with AOM in younger children, but some epidemiologic studies have shown that a universal antibiotic therapy strategy in all cases of AOM cannot be considered a valid way to reduce its risk. While the watchful waiting policy seems to lead to only 1 to 2 extra cases per 100,000 children per year, several studies have concluded that the number of children who would need to be treated to prevent this complication is too high and that this strategy could pose a larger public health problem in terms of antibiotic resistance [7,22-24]. Our study did not find any association between the number of cases of acute mastoiditis and the percentage of patients treated with antibiotics, as the annual rate was found to be stable before and after the new recommendations were published. As the results of some studies appear to be contradictory, it is impossible to draw clear conclusions [25,26]. Finnbogado’ttir, for example, concluded there might be a correlation between reduced antibiotic usage in children in Iceland and an increasing incidence of mastoiditis following changes in the guidelines for antibiotic prescriptions for AOM in that country; another study carried out in Sweden found no increase after new guidelines were introduced [25,26].

We found a stable percentage of antibiotic usage also in the children between 2 and 6, a group in which there was an increase in the number of cases of acute mastoiditis. Forty-two percent of the children who were diagnosed with acute mastoiditis after the guidelines were published were not prescribed antibiotic treatment. Prospective studies could help to elucidate if these findings could be the result of misdiagnosis.

Despite the fact that anatomical and physiological reasons make children younger than 2 more susceptible to AOM, only 32% of the children in that age bracket in the Netherlands presented this complication [27]; in Sweden there was an increase in acute mastoiditis only in children younger than 2 in whom antibiotics were still recommended in all cases of AOM [26].

The limitations of our study include its retrospective design which does not permit an accurate assessment of illness severity/duration and does not provide follow-up data. Management of missing data can also pose methodological problems. In addition, while our research was made using appropriate key words, we cannot exclude that a few cases might have been missed. Data registered by paediatricians on staff may vary in detail and accuracy. Another possible referral bias could be linked to sicker patients with persistent symptoms who are frequently taken to the PED.

Adherence to the guidelines could probably be enhanced if effective information campaigns were implemented. In addition, the watchful waiting approach would probably be utilized more frequently if, at discharge time, parents were given an appointment for a check-up.

## Conclusions

Despite the introduction of clinical guidelines that recommend a watchful waiting approach for children with AOM, antibiotic prescribing rates in an Italian PED are still high. It appears to be more difficult to impact the percentage of cases for which antibiotics are prescribed than the type of antibiotic that is utilized. In view of these findings, a close follow-up control on the infection’s progression and incisive campaigns to promote parents’ awareness of proper antibiotic use appear more than warranted.

## References

[1] Bardach A, Ciapponi A, Garcia-Marti S, Glujovsky D, Mazzoni A, Fayad A (2011). Epidemiology of acute otitis media in children of Latin America and the Caribbean, a systematic review and metanalysis. Int J Pediatr Otorhinolaryngol.

[2] Venekamp RP, Sanders S, Glasziou PP, Del Mar CB, Rovers MM. Antibiotics for acute otitis media in children. Cochrane Database Syst. Rev 2013, Jan 31:1 CD000219.doi.10.1002/14651858.CD000219.pub3 10.1002/14651858.CD000219.pub3 23440776

[3] Paradise JL, Rockette HE, Colborn DK, Bernard BS, Smith CG, Kurs-Lasky M (1997). Otitis media in 2253 Pittsburgh area infants: prevalence and risk factors during the first two years of life. Pediatrics.

[4] Coticchia JM, Chen M, Sachdeva L, Mutchnick S (2013). New paradigms in the pathogenesis of otitis media in children. Frontiers in Pediatrics.

[5] Forgie S, Zhanel G, Robinson J (2009). Management of acute otitis media. Paediatr Child Health.

[6] Stenfeldt K, Hermansson A (2010). Acute mastoiditis in southern Sweden: a study of occurrence and clinical course of acute mastoiditis before and after introduction of new treatment recommendations for AOM. Eur Arch Otorhinolaryngol.

[7] Schilder AGM, Lok W, Rovers MM (2004). International perspectives on management of acute otitis media: a qualitative review. Int J Pediatr Otorhinolaryngol.

[8] Williamson I, Benge S, Mullee M, Little P (2006). Consultation for middle ear disease, antibiotic prescribing and risk factors for reattendance: a case-linked cohort study. Br J Gen Pract.

[9] Haggard M (2011). Poor adherence to antibiotic prescribing guidelines in acute otitis media-obstacles, implications, and possible solutions. Eur J Pediatr.

[10] Barber C, Ille S, Vergison A, Coates H (2014). Acute otitis media in young children-What do parents say?. Int J Pediatr Otorhinolaryngol.

[11] Lieberthal AS, Carroll AE, Chonmaitree T, Ganiats TG, Hoberman A, Jackson MA (2013). The diagnosis and management of acute otitis media. Pediatrics.

[12] Marchisio P, Bellussi L, Di Mauro G, Doria M, Felisati G, Longhi R (2010). Acute otitis media: from diagnosis to prevention. Summary of the Italian Guideline. Int J Pediatr Otorhinolaryngol.

[13] Marchisio P, Cantarutti L, Sturkenboom M, Girotto S, Picelli G, Dona D (2012). Burden of acute otitis media in primary care paediatrics in Italy: a secondary data analysis from the Pedianet database. BMC Pediatr.

[14] Kershner JE, Kliegman RM, Stanton BF, Schor NF, St Geme JW, Behman RE (2011). Otitis media. Nelson textbook of paediatrics.

[15] Fischer T, Singer AJ, Lee C, Thode HC (2007). National trends in emergency department antibiotic prescribing for children with acute otitis media,1996-2005. Acad Emer Med.

[16] Cabana MD, Rand CS, Powe NR (1999). Why don’t physician follow clinical guidelines? A framework for improvement. JAMA.

[17] Sibbald AD (2012). Acute otitis media in infants: the disease and the illness. Clinical distinctions for the new treatment paradigm. Otolaryngol Head Neck Surg.

[18] Chao JH, Kunkov S, Reyes LB, Lichten S, Crain EF (2008). Comparison of Two Approaches to Observation Therapy for Acute Otitis Media in the Emergency Department. Pediatrics.

[19] Steinmann K, Babl FE (2006). Antibiotic prescribing rates for acute otitis media in a paediatric emergency department. J Paediatr Child Haelth.

[20] Levy C, Pereira M, Guedj R, Abt.Nord C, Baudino Gelbert N, Cohen R (2014). Impact of 2011 French guidelines on antibiotic prescription for acute otitis media in infants. Med et Mal Infect.

[21] Celind J, Sodemark L, Hjalmarson O (2014). Adherence to treatment guidelines for acute otiits media in children. The necessity of an effective strategy of guideline implementation. Int J Pediatr Otorhinolaryngol.

[22] Goldstein NA, Casselbrant ML, Bluestone CD, Kurs-Lasky M (1998). Intratemporal complications of acute otitis media in infants and children. Otolaryngol Head Neck Surg.

[23] Gliklich RE, Eavey RD, Iannuzzi RA, Camacho AE (1996). A contemporary Analysis of Acute Mastoiditis. Arch Otolaryngol Head Neck Surg.

[24] Thompson PL, Gilbert RU, Long PF, Saxena S, Sharland M, Wong IC (2009). Effect of antibiotics for otitis media on mastoiditis in children: a retrospective cohort study using the United Kingdom general practice research database. Pediatrics.

[25] Finnbogado’ttir AF, Petersen H, Laxdal R, Gudbrandsson F, Gudnason R, Haraldsson A (2009). An increasing incidence of mastoiditis in children in Iceland. Scand J Infect Dis.

[26] Groth A, Enoksson F, Hermansson A, Hultcrantz M, Stalfors J, Stenfeldt K (2011). Acute mastoiditis in children in Sweden 1993-2007-No increase after new guidelines. Int J Pediatr Otorhinolaryngol.

[27] Van Zuijlen DA, Schilder AG, Balen FA, Hoes AW (2001). National differences in incidence of acute mastoiditis: relationship to prescribing patterns of antibiotics for acute otitis media?. Pediatr Infect Dis J.

